# Multicenter Study Examining Temporal Trends in Traumatic Intracranial Hemorrhage Over Six Years Using Joinpoint Regression

**DOI:** 10.1089/neur.2024.0097

**Published:** 2024-10-09

**Authors:** Timbre Backen, Kristin Salottolo, David Acuna, Carlos H. Palacio, Gina Berg, Andrea Tsoris, Robert Madayag, Kaysie Banton, David Bar-Or

**Affiliations:** ^1^ Trauma Services Department, Swedish Medical Center, Englewood, Colorado, USA.; ^2^ Trauma Research Departments, Swedish Medical Center, Englewood, Colorado, USA.; ^3^ South Texas Health System, McAllen, Texas, USA.; ^4^ Wesley Medical Center, Wichita, Kansas, USA.; ^5^ Trauma Services Department, Wesley Medical Center, Wichita, Kansas, USA.; ^6^ Trauma Services Department, South Texas Health System, McAllen, Texas, USA.; ^7^ Trauma Services Department, Penrose Hospital, Colorado Springs, Colorado, USA.; ^8^ Trauma Services Department, St. Anthony Hospital, Lakewood, Colorado, USA.; ^9^ Trauma Services Department, Lutheran Hospital, Denver, Colorado, USA.

**Keywords:** age, epidemiology, head trauma, surgery, traumatic brain injury

## Abstract

The aging US population has altered the epidemiology of traumatic injury, but there are few studies examining changing patterns of traumatic intracranial hemorrhage (tICH). We examined temporal changes in incidence, demographics, severity, management, and outcomes of tICH among trauma admissions at six US Level I trauma centers over 6 years (July 1, 2016–June 30, 2022). Patients with tICH (subdural, epidural, subarachnoid, and intracerebral hemorrhage) were identified by 10th revision of the International Statistical Classification of Diseases diagnosis codes. Temporal trends were examined over 12 six-month intervals using joinpoint regression and reported as biannual percent change (BPC); models without joinpoints are described as linear trends over time. There were 67,514 trauma admissions over 6 years and 11,935 (17.7%) patients had a tICH. The proportion of tICH injuries significantly increased 2.6% biannually from July 2016 to July 2019 (BPC = 2.6, *p* = 0.04), then leveled off through June 2022 (BPC = −0.9, *p* = 0.19). Similarly, the proportion of geriatric patients (≥65 years old) increased 2.4% biannually from July 2016 to July 2019 (BPC = 2.4, *p* = 0.001) as did injuries due to falls (BPC = 2.2, *p* = 0.01). Three of the four most prevalent comorbidities significantly increased: hypertension linearly increased 2.1% biannually, functional dependence increased 25.5% biannually through June 2019, and chronic anticoagulant use increased 19.0% biannually through June 2019 and then 3.1% thereafter. There were no trends in the rates of neurosurgical intervention (BPC = −0.89, *p* = 0.40), ED Glasgow coma score 3–8 (BPC = −0.4, *p* = 0.77), or presence of severe extracranial injuries (BPC = −0.7, *p* = 0.45). In-hospital mortality linearly declined 2.6% biannually (BPC = 2.6, *p* = 0.05); however, there was a 10.3% biannual linear increase in discharge to hospice care (BPC = 10.3, *p* < 0.001). These results demonstrate the incidence of tICH admissions is temporally increasing, and the population is growing older with more comorbidities and injuries from falls. Yet, traumatic brain injury severity and neurosurgical management are unchanged. The shift from in-patient death to hospice care suggests an increased need for palliative care services.

## Introduction

Traumatic brain injury (TBI) is a major cause of death and disability in the United States. Approximately 190 Americans die each day from TBI-related injuries, and all-cause mortality is increased two-fold after head injury.^1,2^ Numerous studies have demonstrated an increase in TBIs over time. A surveillance summary from the Centers for Disease Control and Prevention (CDC) reported an increase in both the incidence and mortality of TBI from 2007 to 2013.^3^ The CDC report primarily attributed the increase in TBIs to older adults suffering falls, which reflects changes in US population demographics: the number of Americans aged ≥65 grew by 39% between 2010 and 2020.^4^ These rising trends in the geriatric population are also of clinical importance because adults aged ≥65 with TBI are at higher risk for in-hospital mortality compared with their younger counterparts.^5,6^

Traumatic intracranial hemorrhage (tICH), a subset of TBI, includes epidural hematoma, subdural hematoma (SDH), subarachnoid hemorrhage, and intracerebral hemorrhage. A tICH is present in roughly half of all TBIs and is associated with a significantly worse prognosis than nonhemorrhagic brain injury.^7^ Moreover, tICH is a leading cause of mortality following traumatic injury, accounting for 40–50% of all traumatic fatalities.^8^ The morbidity of tICH is due to a multitude of factors. As elucidated by the Monro–Kellie hypothesis, total intracranial volume must remain constant; thus, mass effect due to intracranial bleeding decreases cerebral blood flow, leading to secondary insults including brain ischemia and hypoxia.^9,10^ Additionally, compromise of the blood–brain barrier secondary to trauma allows circulating inflammatory cells to enter the central nervous system which can lead to prolonged neuroinflammation and contribute to morbidity.^11^

Management of tICH can significantly vary depending on the severity of injury. Most organizations suggest baseline medical management including blood pressure control, seizure prophylaxis, and management of intracranial pressure.^12^ The decision to pursue neurosurgical intervention is dependent upon a multitude of factors but largely depends on whether the patient’s outcome may benefit from cranial decompression. Some data support early, aggressive surgical management for tICH, particularly in patients with depressed Glasgow coma scale (GCS) scores on admission.^13^ There remains high levels of variability in the neurosurgical management of TBI across the United States, which may be due to changing hemorrhage characteristics and practice patterns over time.^14^

Trends in tICH are important to differentiate from trends in the broader category of TBI because more than 15% of TBI-related hospitalizations involve an isolated concussion,^15^ and management and outcomes of TBI depend on the presence of tICH. The aim of this study is to examine temporal changes in tICH including incidence, patient demographics, injury severity, and outcomes.

## Methods

### Population

This was a retrospective registry-based cohort study that included adult (age ≥18 years) trauma patients at six US Level I trauma centers from July 1, 2016, to June 30, 2022. There were no exclusion criteria. This study received institutional review board (IRB) approval with a waiver of informed consent from the respective IRBs at each facility: Penrose Hospital, Colorado Springs, CO; STHS-McAllen Hospital, McAllen, TX; Wesley Hospital, Wichita, KS; Swedish Medical Center, Englewood, CO; St. Anthony Hospital, Lakewood, CO; Medical City Plano, Plano, TX. The study was performed in accordance with the ethical standards as laid down in the 1964 Declaration of Helsinki and its later amendments or comparable ethical standards.

### Covariates and outcomes

Covariates and outcomes examined for this study were abstracted from patient charts into the trauma registry by dedicated trauma registrars at all the participating facilities and were collected using standard definitions as outlined in the National Trauma Data Standards dictionary. The trauma registry includes data from time of injury through discharge (or death).

Patients with tICH (subdural, epidural, subarachnoid, and intracerebral hemorrhages) were identified in the registry using the 10th revision of the International Statistical Classification of Diseases (ICD-10) diagnosis codes of S06.4–S06.6 and S06.34–S06.36.

The main exposure variable was hospital arrival date, categorized into biannual periods (January through June and July through December).

Demographic variables were examined as age (18–64 vs. ≥65 years), sex (male vs. female), race (Hispanic, non-Hispanic [NH] White, NH-Black, and other NH race), comorbidities (with ≥10% incidence), and fall cause of injury (vs. other causes). Injury severity was examined as neurosurgical management (yes/no), emergency department (ED) GCS (3–8 vs. 9–15), and severe extracranial injury (any region other than the head/neck with an abbreviated injury scale [AIS] score ≥3). Neurosurgery was defined using ICD-10-PC procedure codes for excision and evacuation (e.g., craniotomy and craniectomy), drainage and monitoring devices (e.g., intracranial pressure monitor and ventriculostomy), and skull repair/resection (e.g., cranioplasty). Hospital discharge disposition was examined as morgue (in-hospital mortality), hospice care, acute care facility (long-term acute care, skilled nursing facility, rehabilitation facility), and home/home health. The aforementioned covariates and outcomes were defined *a priori*, while presumed death was defined *a posteriori* as hospital discharge disposition to the morgue or hospice.

### Statistical analysis

Joinpoint models were used to examine temporal changes over biannual time periods in tICH incidence, as well as temporal changes in patients with tICH by demographic characteristics (age, sex, race, cause of injury, comorbidities), severity characteristics (neurosurgery, GCS, extracranial injury) and discharge disposition. A joinpoint regression model segments time series data into groups of data points with similar linear trends to identify inflection points (i.e., joinpoints); this type of analysis differs from linear regression or Cochran–Armitage trend tests that assume linearity over the entire time series to fit a “best” straight line.

First, SAS® version 9.4 was used to summarize the proportion of patients in each biannual time period with tICH as well as proportions of the tICH population based on patient demographics, severity characteristics, and discharge disposition. Next, the proportion point estimates and their standard errors were analyzed over time via joinpoint regression models using the National Cancer Institute joinpoint software program version 5.0.2^16^ and the methods proposed by Kim et al.^17^ A linear model with zero joinpoints was initially fit to the data and the change over time was examined as biannual percent change (BPC). Additional joinpoints were added when the slope of the line between joinpoints was significantly different from zero (*p* < 0.05 based on the BPC compared to zero). A stable or nonsignificant trend was defined based on a *p* ≥ 0.05 when comparing the BPC with zero. The maximum number of joinpoints for our dataset was 3 based on 12 data points in our series. The minimum weighted Bayesian information criterion method was used to determine the number of joinpoints in the final selected model (Joinpoint 5.0 default setting). Models without joinpoints are described as linear trends over time.

## Results

There were 67,514 trauma admissions over 6 years and 11,935 (17.7%) patients had a tICH diagnosis. The most common diagnosis was a SDH (66.5%) and nearly all tICH (98.4%) resulted from blunt mechanism of injury, with the most common cause of tICH due to falls (65.5%) (Table 1). Most patients were male (60.0%), NH-White (75.8%), and ≥65 years old (54.5%). The most prevalent comorbid conditions were hypertension (45.9%), diabetes (17.9%), chronic anticoagulant use (14.6%), and functional dependence (14.2%). Overall, 12.4% of patients had neurosurgical intervention. The overall rate of in-hospital death was 8.5%, which represented 48.7% of all trauma fatalities.

**Table 1.** Descriptive Characteristics of Trauma Admissions by Traumatic Intracranial Hemorrhage Diagnosis

Covariate, *n* (%)	tICH diagnosis (*n* = 11,935)	No tICH (*n* = 55,579)
Age ≥65 years	6505 (54.5)	24,434 (44.0)
Female sex	4771 (40.0)	24,936 (44.9)
Non-Hispanic White	9048 (75.8)	41,253 (74.2)
Non-Hispanic Black	433 (3.6)	2704 (4.9)
Hispanic	1640 (13.7)	8534 (15.4)
Other non-Hispanic race	814 (6.8)	3088 (5.6)
Fall cause of injury	7819 (65.5)	28,955 (52.1)
Vehicular cause of injury	1482 (12.4)	9226 (16.6)
Assault cause of injury	512 (4.3)	2952 (5.3)
Other cause of injury	1612 (13.5)	11,969 (21.5)
Hypertension comorbidity	5479 (45.9)	20,003 (36.0)
Dementia comorbidity	1286 (10.8)	4221 (7.5)
Anticoagulant use comorbidity	1745 (14.6)	5205 (9.4)
Diabetes comorbidity	2138 (17.9)	7459 (13.4)
Functionally dependent	1689 (14.2)	6864 (12.4)
Current smoker comorbidity	1626 (13.6)	9577 (17.2)
Subdural hematoma	7936 (66.5)	0 (0)
Epidural hematoma	540 (4.5)	0 (0)
Subarachnoid hemorrhage	6347 (53.2)	0 (0)
Intracerebral hemorrhage	2151 (18.0)	0 (0)
Neurosurgery	1480 (12.4)	0 (0)
Discharge to hospice	461 (3.9)	419 (0.7)
Discharge to home	6323 (53.0)	33,940 (61.4)
Discharge to care facility	3672 (30.8)	17,383 (31.4)
In-hospital mortality	1017 (8.5)	1072 (1.9)
Presumed death (morgue/hospice)	1478 (12.4)	1491 (2.7)

Data were obtained from trauma registries of six US Level I trauma centers, July 1, 2016–June 30, 2022. Chi-square statistics for comparison of proportions by group (tICH vs. no tICH) yielded *p* values <0.001 for all covariates.

tICH, traumatic intracranial hemorrhage.

**FIG. 1.**
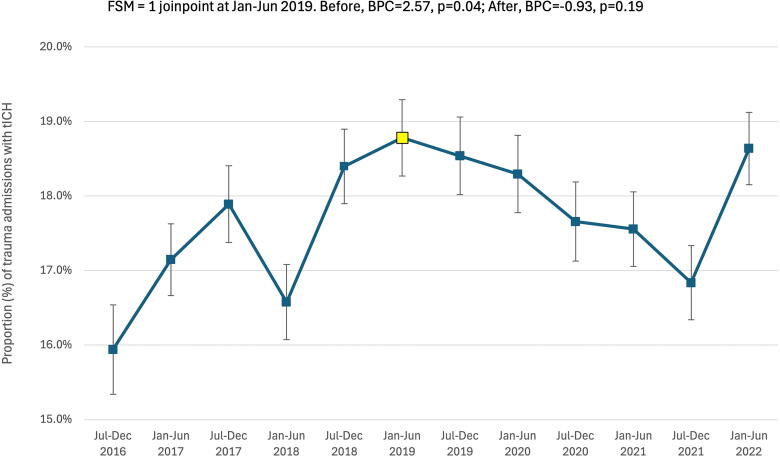
Temporal changes in traumatic admissions with tICH. Joinpoints are denoted with red and yellow data points. Data were obtained from trauma registries of six US Level I trauma centers. BPC, biannual percent change; FSM, final selected model; tICH, traumatic intracranial hemorrhage.

The final model examining tICH incidence over time demonstrated a significant 2.6% biannual increase in tICH between July–December 2016 and January–June 2019 (BPC = 2.6, *p* = 0.04), followed by a leveling off of tICH injuries through June 2022 (BPC = −0.9, *p* = 0.19) (Figure 1). The absolute number of tICH admissions also peaked around January–June 2019, with 1052 biannual admissions for tICH.

**FIG. 2.**
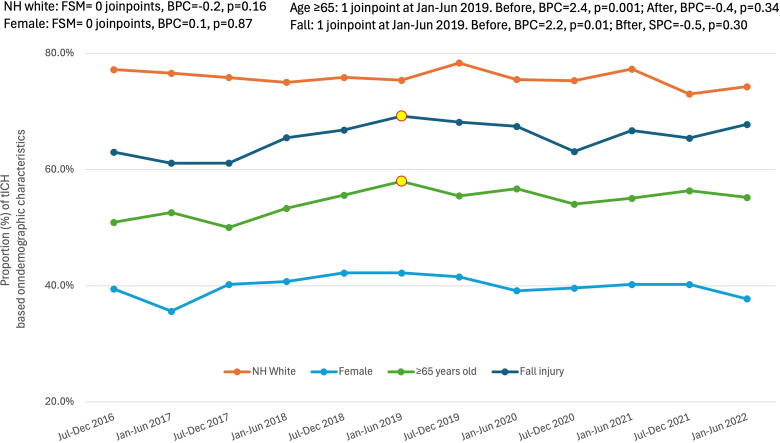
Temporal change in demographics for patients admitted with tICH. Joinpoints are denoted with red and yellow data points. Data were obtained from trauma registries of six US Level I trauma centers. BPC, biannual percent change; FSM, final selected model; tICH, traumatic intracranial hemorrhage.

Temporal changes in demographics are shown in Figure 2. The model examining the proportion of geriatric patients (≥65 years old) with tICH significantly increased 2.4% biannually from July 2016 through January–June 2019 (BPC = 2.4, *p* = 0.001), then leveled off through June 2022 (BPC = −0.4, *p* = 0.34). Likewise, a fall cause of injury significantly increased 2.2% biannually from July 2016 through January–June 2019 (BPC = 2.2, *p* = 0.01), which then leveled out through June 2022 (BPC = −0.5, *p* = 0.30). There were no temporal trends in sex (BPC = −0.1) or NH-white race (BPC = −0.8) for patients with tICH over the 6-year period. There was a significant 4.6% biannual linear increase in the proportion of NH-Black patients with tICH injuries over time (BPC = 4.6, *p* = 0.03; data not plotted).

**FIG. 3.**
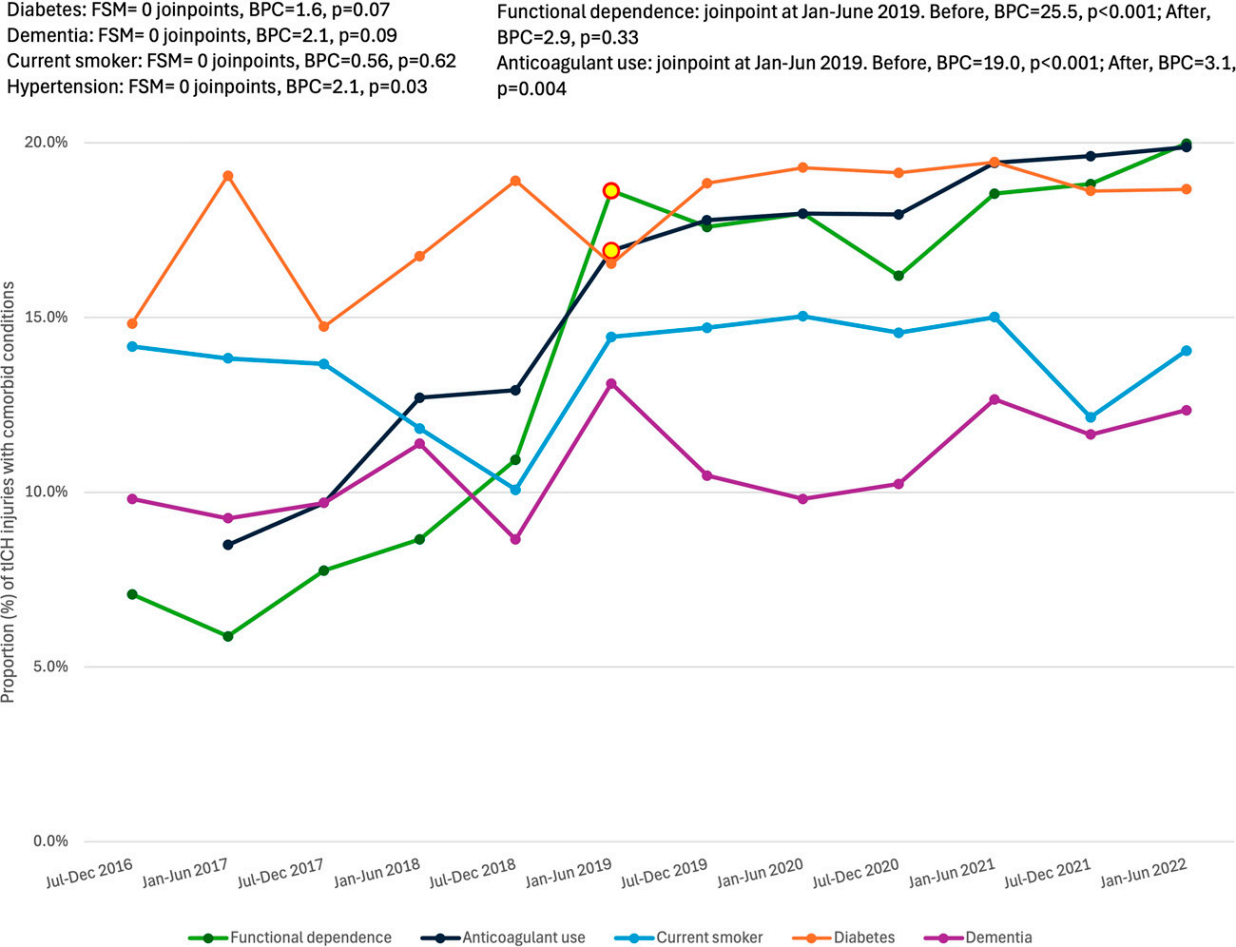
Temporal change in prevalent comorbidities for patients admitted with tICH. Joinpoints are denoted with red and yellow data points. Data were obtained from trauma registries of six US Level I trauma centers. Comorbidities with at least 10% prevalence in the tICH population were analyzed. BPC, biannual percent change; FSM, final selected model; tICH, traumatic intracranial hemorrhage.

Figure 3 displays the proportion of tICH patients with a prevalent comorbidity and trends in comorbidities over time. For functional dependence, there was a 25.5% biannual increase from July 2016 through January–June 2019 (BPC = 25.5, *p* < 0.001), which leveled out through June 2022 (BPC = 2.9, *p* = 0.33). For chronic anticoagulant use, there was a 19.0% biannual increase from July 2016 through January–June 2019 (BPC = 19.0, *p* < 0.001), and a further 3.1% increase through June 2022 (BPC = 3.1, *p* = 0.004). The models examining hypertension, dementia, diabetes, and smoking demonstrated 0 joinpoints: hypertension linearly increased 2.1% biannually (*p* = 0.03, data not plotted), whereas rates of dementia, diabetes, and smoking status did not change over time.

**FIG. 4.**
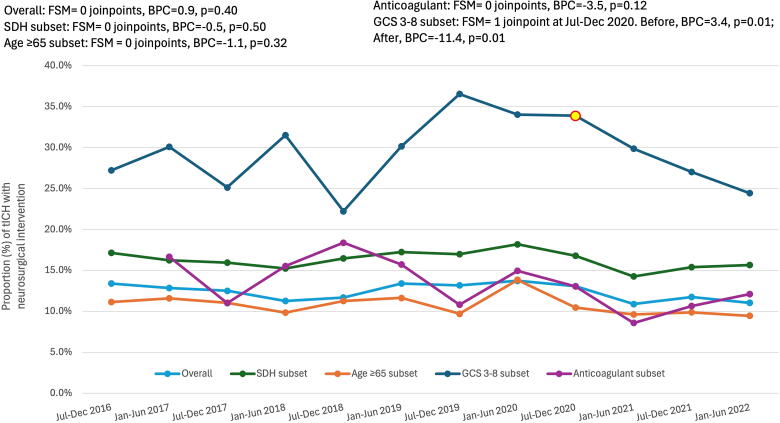
Temporal change in neurosurgical management for patients admitted with tICH. Joinpoints are denoted with red and yellow data points. Data were obtained from trauma registries of six US Level I trauma centers. BPC, biannual percent change; FSM, final selected model; tICH, traumatic intracranial hemorrhage.

There were no trends in injury severity as assessed by an ED GCS 3–8 (BPC = −0.4, *p* = 0.77) or in the presence of an extracranial injury (AIS ≥ 3 to a region other than the head/neck; BPC = −0.7, *p* = 0.45). As shown in Figure 4, there were also no trends in neurosurgical intervention over the 6-year study period (BPC = −0.89, *p* = 0.40). When examining neurosurgical management in patient subsets, there were also no trends in neurosurgery in geriatric patients (BPC = −1.1, *p* = 0.32), in patients with SDH (BPC = −0.5, *p* = 0.50) or in patients on chronic anticoagulants (BPC = −3.5, *p* = 0.12). In patients with ED GCS 3–8, there was one joinpoint identified at July–December 2020, with a significant increase in neurosurgery before the joinpoint (BPC = 3.4, *p* = 0.01), and a significant decline in neurosurgery after the joinpoint (BPC = −11.4, *p* = 0.01; Fig. 4).

**FIG. 5.**
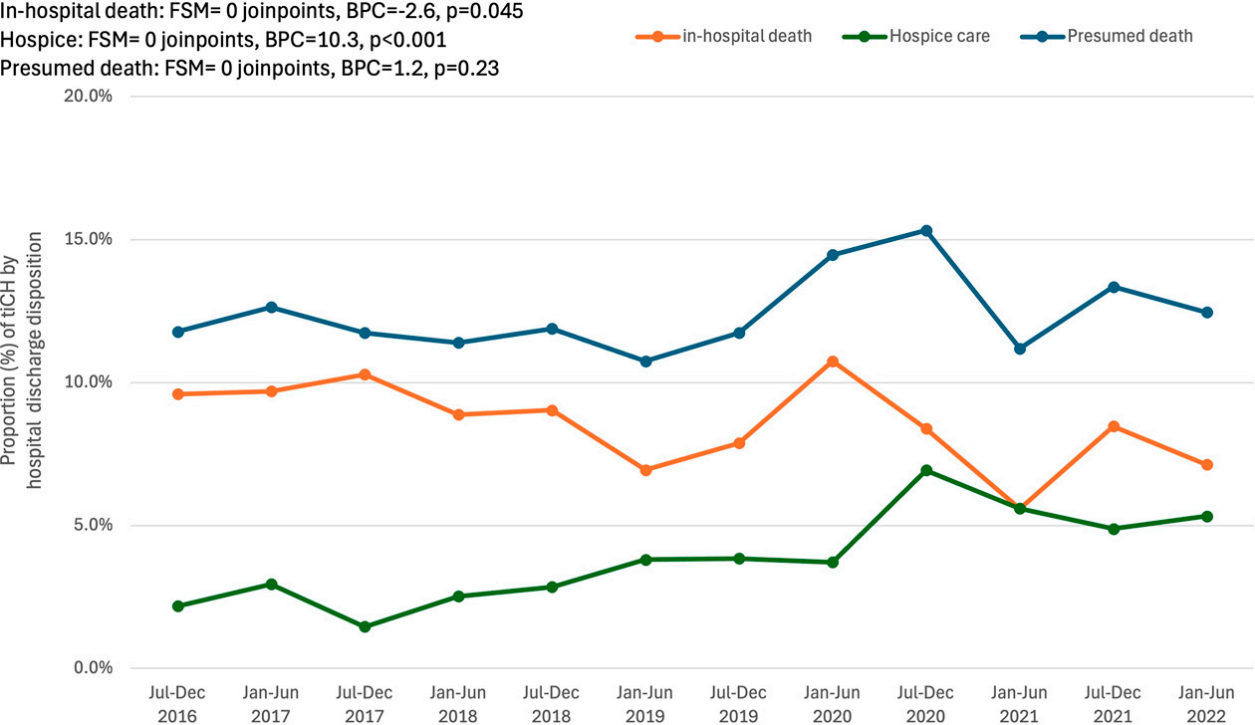
Temporal change in-hospital discharge disposition for patients admitted with Data were obtained from trauma registries of six US Level I trauma centers. BPC, biannual percent change; FSM, final selected model; tICH, traumatic intracranial hemorrhage.

As shown in Figure 5, there was a significant 2.6% biannual linear decline in in-hospital mortality (BPC = 2.6, *p* = 0.045); however, there was also a significant 10.3% biannual linear increase in discharge to hospice care (BPC = 10.3, *p* < 0.001). When combined, the rate of presumed death (discharge to hospice or morgue) was unchanged over time (BPC = 1.2, *p* = 0.23). There was no change in discharge to home/home health care (BPC = 0.7, *p* = 0.90), and a small but significant 1.9% linear decline in discharge to a care facility (BPC = −1.9, *p* = 0.01, data not plotted).

## Discussion

To our knowledge, our study is the first to evaluate temporal trends in tICH. This epidemiological study of six Level I trauma centers suggests that tICH is becoming more prevalent and is being driven by a shift to older, frailer patients injured from falls.

As the US population ages over time, a greater proportion of tICH is occurring in elderly patients. There was a 14% increase in tICH admissions among patients ≥65 years old from July 2016 to July 2019 (from 51% to 58% of all tICHs). This is significant to health care providers and hospital systems, as the practice of geriatric medicine is associated with unique considerations. Furthermore, we saw an increase in patient comorbidities including hypertension, functional dependency, and especially chronic anticoagulant use, which doubled from 8.5% in 2017 to 19.9% in 2022. Several studies have found a positive correlation between anticoagulant or antiplatelet use and death after tICH.^18,19^ Thus, the 19% biannual increase in anticoagulation use among admitted patients with tICH should be a significant concern to emergency room physicians, trauma surgeons, and neurosurgeons. Nederpelt et al. examined geriatric patients admitted with tICH due to a fall between 2011 and 2018, and anticoagulated patients were significantly more likely to undergo neurosurgical intervention.^20^

Our findings are similar to data published by the CDC on trends in TBI-related hospitalizations from 2007 to 2013 regarding increases in elderly patients hospitalized with TBI after falls.^3^ However, the CDC report did not demonstrate any trend in the total number of all-cause TBI admissions from 2007 to 2013, whereas we found an increase in the proportion of tICH admissions from July 2016 to July 2019. This difference may be multifactorial but could be due to a greater proportion of fall injuries in our population (66%) compared with the CDC hospitalization data (50%) or this could suggest that tICH diagnoses are increasing and concussions are decreasing (yielding no net change in TBI generally) or that there are higher thresholds for inpatient management of nonhemorrhagic and mild TBI and thus a greater proportion of all admitted TBIs are tICH. Alternatively, these data might suggest that there was an acceleration in tICH (and potentially TBI) admissions between 2016 and 2019 that has not been previously reported, which might be related to the growing elderly population and the increased use of antithrombotic medications.

There was a trend of greater neurosurgical intervention in patients with ED GCS 3–8, where the rate of neurosurgery increased from 27% to 37% between July 2016 and July 2019 before declining to 24% through June 2022. Conversely, in patients with mild tICH (ED GCS 13–15), a retrospective national registry review reported a significant decline in neurosurgical intervention over time from 2007 to 2019.^14^ These seemingly disparate findings could be explained by the implementation of Brain Injury Guidelines (BIG).^21^ The BIG guidelines offer a stratified algorithm to guide the management of tICH with the intention of encouraging improved usage of resources, such as neurosurgical monitoring of patients with severe TBI (GCS 3–8 after resuscitation) and abnormal CT scan (e.g., tICH) and reduced ICU admission for patients with mild neurological deficits. Interestingly, there was no change in the rate of neurosurgical intervention overall, or in patient subsets of age ≥65 years, an SDH diagnosis, or chronic anticoagulant use, potentially due to an increased availability of anticoagulant reversal agents or a shift away from aggressive surgical management of elderly patients with expected poor postoperative quality of life.

Several regression models identified the same joinpoint, in January–June 2019, representing a high degree of collinearity between dependent variables such as geriatric age, presence of comorbidities, and fall cause of injury. While we were not surprised that the same joinpoint was identified for multiple covariates, we do not have an explanation as to why this particular time point, January–June 2019, saw a reversal in the rising trend of tICH diagnoses for older, frailer patients injured from a fall. The national rise in fall prevention programs implemented in nursing homes and hospital settings occurred prior to 2019, including the 2012 launch of the Stopping Elderly Accidents, Deaths, and Injuries initiative by the CDC and the 2015 Falls Free® National Falls Prevention Plan.^22,23^ More research is needed to determine if the trends we observed are generalizable to other trauma systems in the United States.

Discharge disposition of tICH shifted over time from in-hospital mortality to discharge to hospice care. Even with the increase in patients discharged to hospice, tICH still accounted for nearly 50% of all in-patient trauma deaths. This temporal shift from inpatient death to hospice care for trauma patients with tICH is important to recognize and has implications for hospital resources, suggesting an increased need for palliative care services for patients with tICH. Hospice and palliative care utilization is on the rise.^24^ However, there is variability in hospice and palliative care rates for geriatric patients, which were used less frequently for trauma patients than for medical patients.^25^ In a study of geriatric patients with TBI, 61% of patients either died in the hospital or within 30 days of discharge, yet only 17.6% had palliative care consultations and 2% were enrolled in hospice.^26^ The significant increase in hospice admissions for patients with tICH from 2016 to 2022 demonstrates a beneficial evolution in end-of-life care over the time period. Further research into the application of hospice resources and subsequent outcomes is necessary.

Generalizability is a major limitation of this study; our data are from six Level I trauma centers and these findings may not be generalizable to trends observed in lower-level trauma centers or nontrauma centers. Next, this was a retrospective study using existing registry data and we were unable to examine outcomes and covariates not collected in the registry, for instance, long-term and functional outcomes. Additionally, anticoagulant use was not collected in 2016, leaving only 11 data points and up to one joinpoint, as compared with the remaining models that had 12 data points and up to two joinpoints. These and other limitations should be considered when interpreting the results of this study.

## Conclusion

Hospitalization for tICH became more prevalent during this 6-year study period, and there was an increased proportion of geriatric patients with comorbidities being admitted, which is reflective of the temporal changes seen in the general population of the United States. The geriatric population typically has a more complicated clinical picture and utilizes greater resources. Patients with tICH also have high morbidity and mortality which poses specific challenges such as needing a multidisciplinary approach to care and a high utilization for end-of-life services. Hospitals may need to adapt to these changing trends to best serve the needs of this vulnerable population.

## Disclaimer

(1) The views, opinions, findings, and conclusions or recommendations expressed in this article are strictly those of the author(s). They do not necessarily reflect the views of South Texas Health System (STHS), its affiliates, or its parent company. STHS takes no responsibility for any errors or omissions in, or for the correctness of, the information contained in this article. (2) This research was supported (in whole or in part) by HCA Healthcare and/or an HCA Healthcare affiliated entity (Swedish Medical Center, Wesley Medical Center, Medical City Plano). The views expressed in this publication represent those of the author(s) and do not necessarily represent the official views of HCA Healthcare or any of its affiliated entities.

## Abbreviations Used

AIS: abbreviated injury scale
BIG: Brain Injury Guidelines
BPC: bi-annual percent change
CDC: Centers for Disease Control and Prevention
ED: emergency department
GCS: Glasgow coma scale
ICD-10: International Statistical Classification of Diseases
IRB: institutional review board
NCI: National Cancer Institute
NH: non-Hispanic
SDH: Subdural hematoma
TBI: Traumatic brain injury
tICH: Traumatic intracranial hemorrhage
